# Progress of porous tantalum surface-modified biomaterial coatings in bone tissue engineering

**DOI:** 10.1007/s10856-025-06871-w

**Published:** 2025-03-05

**Authors:** Aiguo Liu, Chenxu Wang, Ziwen Zhao, Rui Zhu, Shuang Deng, Sitong Zhang, Farnaz Ghorbani, Ting Ying, Chengqing Yi, Dejian Li

**Affiliations:** 1 Department of Orthopedics, The First Affiliated Hospital of Henan University, Kaifeng, China; 2 Department of Orthopedics, Shanghai Pudong Hospital, Fudan University Pudong Medical Center, Shanghai, China; 3 Department of Orthopedics, The First Affiliated Hospital of Harbin Medical University, Harbin, China; 4 Shanghai YangZhi Rehabilitation Hospital (Shanghai Sunshine Rehabilitation Center), School of Medicine, Tongji University, Shanghai, China; 5 Department of Translational Health Sciences, University of Bristol, Bristol, UK

## Abstract

Tantalum (Ta) metal has emerged as a prominent material within the realm of bone tissue engineering, owing to its favorable biocompatibility, commendable mechanical attributes, and notable biological properties such as osteoconductivity, osteoinductivity, and angiogenic potential. However, as clinical applications have expanded, Ta implants have unveiled a spectrum of limitations. Consequently, porous tantalum (PTa) has garnered escalating interest, attributable to its unique microstructural attributes, tunable mechanical characteristics, and inherent biocompatibility. Various methodologies have been proposed to modify the surface of PTa, with the aim of accelerating and enhancing osseous integration while fostering more robust osseointegration. Strategic surface modifications have the potential to augment the inherent advantages of PTa, thereby offering diverse avenues for exploration within the realm of surface effects on PTa. This review elucidates the ongoing research endeavors concerning diverse biomaterial coatings applied to PTa surfaces in the context of bone tissue engineering.

**Graphical Abstract:**

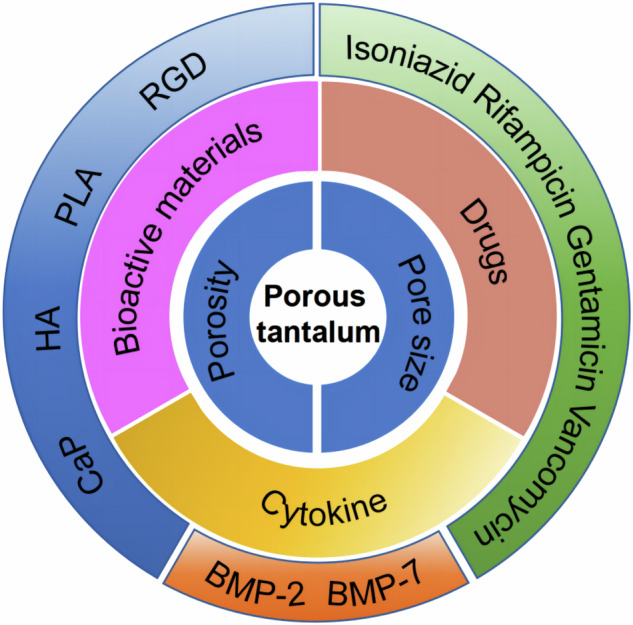

## Introduction

Bone defects are an increasingly common clinical problem of partial skeletal loss caused by severe limb trauma, bone infections, tumors, and osteomyelitis, resulting in a significant reduction in quality of life for millions of patients each year [1, 2]. When a bone defect exceeds the self-healing scale of bone, the body is unable to repair it on its own, and it is necessary to intervene in the treatment by implanting a bone repair material at the site of the defect [3, 4]. Currently, commonly used restorative materials include autogenous bone, allogeneic bone, and artificial bone restorative materials, of which autogenous bone is considered the “gold standard” for bone reconstruction, but the limited donor area and the tendency to cause damage to the donor area and other complications limit its application [5]. Allogeneic bone grafts are prone to immune rejection, cross-infection and other related complications [6]. The utilization of artificial materials for bone repair is constrained by deficiencies in mechanical integrity [7]. Therefore, finding more suitable bone replacement materials has been the focus and hotspot in the field of tissue engineering research [8, 9].

Metals stand as particularly advantageous materials for addressing bone defects through implantation, owing to their abundant material reservoirs, commendable mechanical characteristics, and favorable biocompatibility profiles. Currently, Ta metal is a widely used metal material in clinical applications due to its good biocompatibility, suitable mechanical properties, excellent osteoconductivity, osteoinductivity and vascularity [10, 11]. However, with more in-depth clinical applications, Ta implants have gradually revealed a series of shortcomings, such as the high density and high melting point of Ta leading to poor processing performance, while the stress shielding effect, surface bioinertness, high cost and other shortcomings also limit its wide application [12]. In contrast, PTa, whose structure is similar to cancellous bone in the human body, has received increasing attention due to its special microstructure, adjustable mechanical properties and good biocompatibility [13, 14].

**Fig. 1:**
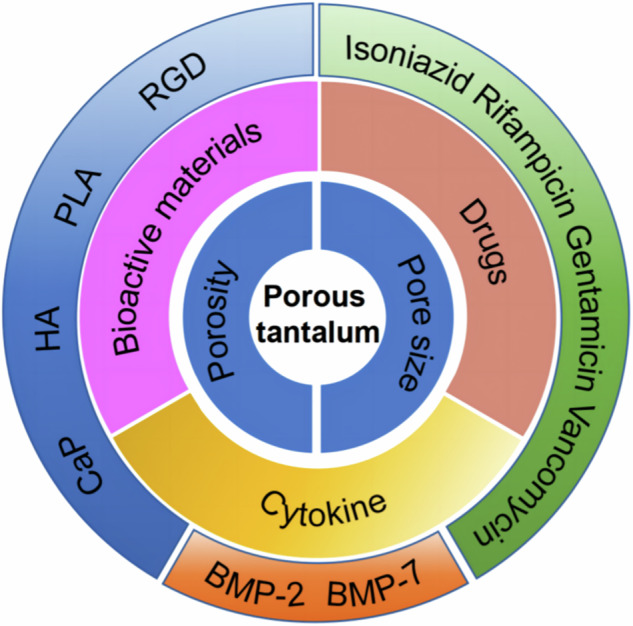
Schematic representation of the functional integration of porous tantalum in biomedical applications

The introduction of PTa into the human body initiates a cascade of interactions between the material surface and the surrounding microenvironment. The dynamics of this interplay are primarily dictated by the surface topographical attributes and the chemical composition inherent to the implant surface [15]. Numerous methodologies have been posited for the alteration of PTa surfaces, with the aim of expediting and enhancing bone ingrowth kinetics, thereby fostering augmented and sustained osseointegration [16]. Strategic surface modifications have the potential to enhance the inherent benefits of PTa, thereby offering diverse avenues for investigating the effects of surface alterations on PTa characteristics. In this paper, the research progress of different biomaterial coatings on PTa surfaces in bone tissue engineering is reviewed, which provides a corresponding reference for PTa implants (Fig. 1).

## Advantages of Ta metal in bone tissue engineering

Ta metal has emerged as a prominent material within the realm of bone tissue engineering, owing to its favorable biocompatibility, commendable mechanical attributes, and notable biological properties such as osteoconductivity, osteoinductivity, and angiogenic potential. Ta exhibits exceptional biocompatibility, which is crucial for minimizing adverse reactions in the body. Its inert nature reduces the risk of inflammation and rejection, making it a favorable choice for long-term implantation [10]. Ta has demonstrated superior osteoconductive and osteoinductive properties, promoting bone cell attachment and proliferation. This is particularly beneficial in enhancing osseointegration, which is essential for the stability and longevity of implants [11]. The unique porous architecture of ta mimics the structure of cancellous bone, allowing for better bone ingrowth and integration. This porosity not only facilitates nutrient and waste exchange but also helps in load transfer, reducing the risk of stress shielding—a common issue with denser metals [14]. Ta possesses mechanical properties that can be tailored to match those of natural bone. Its modulus of elasticity is closer to that of cancellous bone compared to titanium, which helps in minimizing stress shielding and promoting healthy bone remodeling around the implant [17]. Ta can enhance angiogenesis, which is vital for the healing process and integration of the implant with the surrounding tissue. This property is particularly advantageous in complex bone defects where vascularization is critical [10]. Ta exhibits excellent corrosion resistance, which is essential for maintaining the integrity of the implant over time. This characteristic helps prevent the release of metal ions into the surrounding tissue, further enhancing biocompatibility [11].

## Application of PTa in bone tissue engineering

Given its exemplary attributes as a biomedical metallic substance, PTa finds extensive utility within the domain of bone tissue engineering, encompassing a broad spectrum of applications. Since the first report of Ta being fabricated into Ta plates for fracture repair surgery in 1940 [18], Ta has been widely used in bone tissue engineering, such as Ta rods [19–21], spinal fusion cages [22, 23], dental implants [24–26], and artificial joints [27–31], with long term clinical evidence demonstrating its use as a surgical materials.

The modulus of elasticity inherent to solid Ta, measured at 186 GPa, substantially surpasses that of native human cortical bone, ranging between 12 and 18 GPa, as well as cancellous bone, ranging between 0.1 and 0.5 GPa. This considerable dissonance in elasticity moduli may precipitate a stress-shielding phenomenon, thereby engendering bone resorption proximal to the Ta implant site, ultimately culminating in implant loosening and detachment [17]. The advent of PTa materials offers a resolution to these challenges, mirroring the porous architecture inherent to natural bone. By augmenting its porosity, the modulus of elasticity and strength inherent to Ta can be attenuated, thereby positioning its elasticity modulus within a range (2.4–3.9 GPa) that bridges the disparity between human cortical bone (12–18 GPa) and cancellous bone (0.1–0.5 GPa). This intermediate modulus fosters a milieu conducive to the preservation of peri-implant bone density and mitigation of long-term bone resorption surrounding the implant site [17, 32]. This also implies that its pore structure and pore size can be regulated by adjusting the preparation process to meet the mechanical demands of bone tissue at different sites.

## The spatial structure of PTa

### Pore size

Porous scaffolds serve as conduits for the transportation of vital nutrients and metabolic byproducts, facilitate cellular proliferation and differentiation, mediate intercellular signaling, and promote capillary network formation via the mechanisms of fluidic diffusion and osmosis. Consequently, the pore dimensions of porous scaffolds intricately modulate cellular behaviors and bone growth dynamics, albeit with the caveat that the optimal pore size manifests heterogeneity across distinct cell populations [33]. It has been shown that porous structures with pore sizes ranging from 150 to 1000 μm can promote bone growth; pore sizes larger than 300 μm can form vascularized bone tissue within them; pore sizes of 50 to 150 μm lead to bone-like growth; and porous structures with pore sizes of less than 50 μm can only form fibrous tissues [34]. Murphy et al. elucidated that cell adhesion exhibits an inverse correlation with pore size escalation, identifying an optimal pore dimension within the porous architecture, specifically at 96 μm, conducive to cellular proliferation [35]. Furthermore, their findings underscored maximal cell adhesion affinity on scaffolds characterized by the smallest pore size (96 μm). Roosa et al. deduced that pore dimensions exceeding 300 μm are requisite for fostering optimal bone regenerative processes [36].

Too large or too small a pore size will have a negative impact on the bone repair ability. Excessive pore dimensions engender an enlarged spatial separation between the osseous tissue and the porous scaffold interface, thereby impeding optimal bone tissue regeneration. Conversely, overly diminutive pore sizes precipitate stress shielding phenomena, accentuating modulus discrepancies between the implant and the host bone, consequently predisposing to host bone resorption and implant failure [33]. An appropriate pore size can be close to the stiffness of the surrounding bone, allowing the implant to effectively transfer loads and mitigate the stress shielding effect. Luo et al. fabricated PTa constructs featuring discrete pore dimensions ranging from 100–200, 200–400, 400–600, to 600–800 μm, respectively. Subsequent in vivo and in vitro investigations delineated that PTa scaffolds characterized by pore sizes within the 400–600 μm range exhibited augmented cellular adhesion, proliferation, and osteogenic differentiation. Additionally, these scaffolds demonstrated superior efficacy in facilitating bone ingrowth and integration processes [37].

### Porosity

Porosity stands as a pivotal determinant for in vivo tissue anchorage, facilitating intimate interfacing between the implant and host tissue milieu. It is widely acknowledged that porosity constitutes the primary factor influencing both the mechanical attributes, including stiffness and rigidity, and the biological characteristics of the implant. While other parameters, such as pore geometry, exert notable secondary effects, porosity remains paramount in governing the overall performance of the implant [38]. Elevated porosity engenders a dual effect: firstly, it diminishes the mechanical robustness of the metallic scaffold, thereby aligning its elastic modulus more closely with that of bone, thereby mitigating stress shielding phenomena. Secondly, heightened porosity affords an expansive specific surface area, fostering enhanced cellular migration and nutrient diffusion, thereby augmenting osseointegration dynamics [39]. Wauthle et al. fabricated a PTa implant characterized by a porosity level of 80%, subsequently substantiating through in vitro and in vivo assessments its commendable osteoconductive qualities and mechanical resilience [40]. Although high porosity structures are not entirely favorable for osteoblast anchoring, the channels may allow for more cellular penetration in vivo and in vitro, and in any case, cells may adhere to the channel boundaries [41]. The cancellous structure of human bone exhibits intricate morphological irregularities, characterized by a porosity range spanning 50% to 90% and pore diameters typically falling within the range of 300–500 μm, characterized by non-uniform anisotropy [42]. PTa implants, which have demonstrated efficacy in clinical applications, exhibit a trabecular architecture characterized by porosity levels ranging from 70 to 85% and an average pore size falling within the spectrum of 400–600 μm [43].

**Fig. 2:**
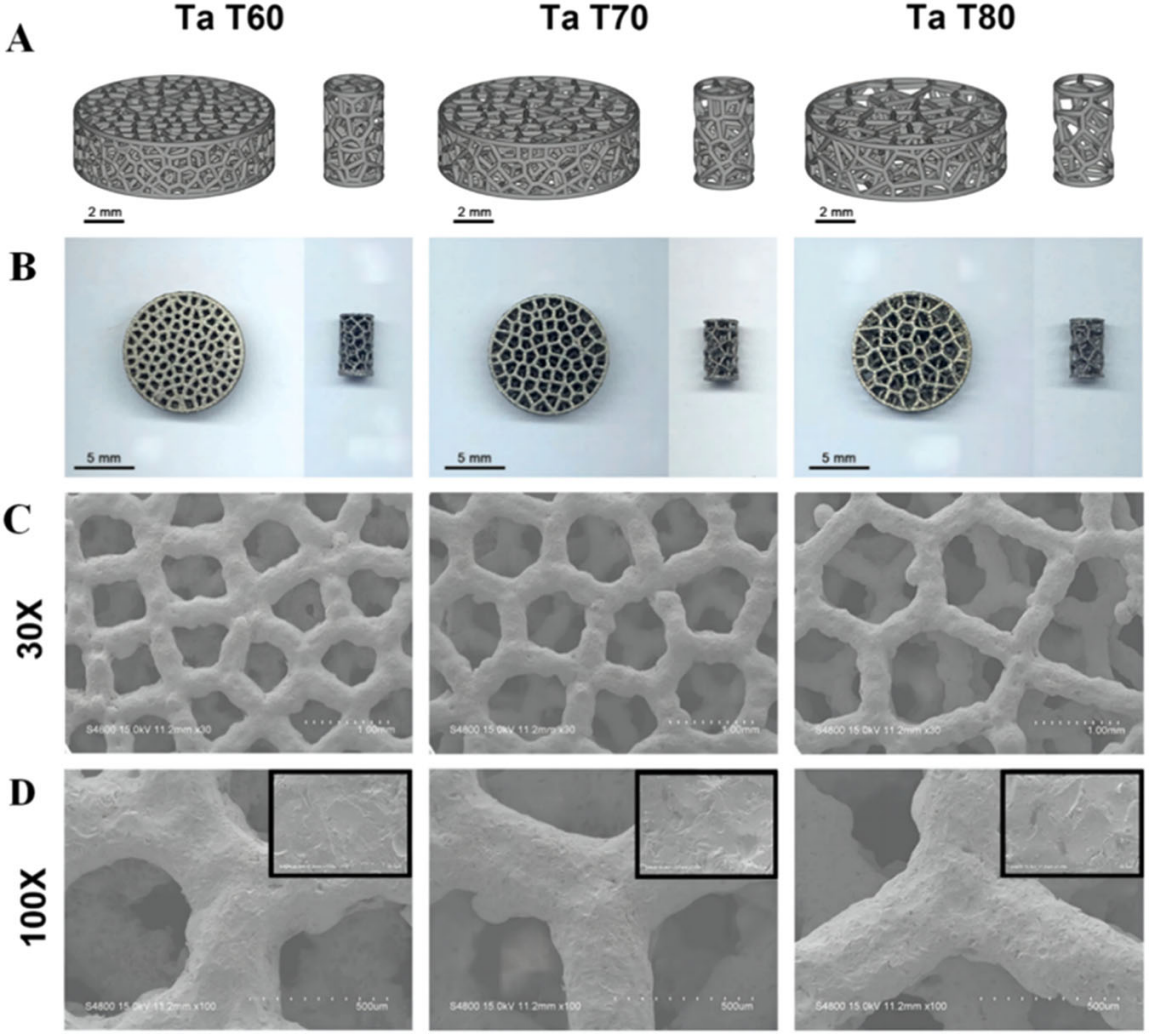
Material characterization of three PTa scaffolds with different porosities. **A** 3D modeling. **B** general appearance (left: disks, right: cylinders). The surface morphology of PTa scaffolds under electron microscopy **C** 30X and **D** 100X magnification (The upper right corner of (**D**) is 1000x magnification) [41]. Reproduced under the terms of the Creative Commons Attribution 4.0 International (CC BY 4.0) license. Copyright © 2023 Jiao, Hong, Zhang, Wang, Tang, Yang, Qu and Yue

Jiao et al. systematically prepared and evaluated three distinct PTa scaffolds featuring varying degrees of porosity (60%, 70%, and 80%) to discern and juxtapose their respective impacts on rat mesenchymal stem cell adhesion, proliferation, and osteogenic differentiation capacities. Their findings elucidated that scaffolds characterized by porosities of 70% and 80% exhibited enhanced osteogenic proliferation and differentiation capabilities, as well as superior bone ingrowth potential compared to those with 60% porosity. Furthermore, in terms of osseointegration, the scaffold with 70% porosity demonstrated preeminence, thereby suggesting the potential superiority of PTa scaffolds featuring a 70% porosity threshold for optimal implant design [41] (Fig. 2). Harmonizing the mechanical integrity and biological functionality of PTa through judicious adjustment of pore ratios stands as a pivotal frontier for future research and development endeavors [44]. The prevailing consensus within the field acknowledges that scaffolds characterized by an average pore diameter of 400 μm and a porosity level of 70% manifest a propensity to facilitate cell migration, proliferation, osteogenic differentiation, as well as the genesis of vascular and osseous tissue [45].

### Modification of biomaterial coatings on PTa surfaces

Owing to Ta’s inherent biocompatibility coupled with its intricate three-dimensional porous configuration, bone tissue exhibits a capacity for regeneration not only on the scaffold’s surface but also within its porous matrix, thereby exemplifying the phenomenon of osseointegration. This phenomenon underscores the bone’s ability to regenerate and integrate both at the interface with the implant’s surface and within its internal structure [46, 47]. The extent of bone ingrowth holds paramount significance in dictating the functional efficacy of PTa implants within an in vivo milieu. Osseointegration, representing the direct structural fusion between bone tissue and the implant surface, emerges as a pivotal determinant not only in securing the stability of the implant but also in safeguarding the overall success of the implantation procedure [48]. Nevertheless, it is noteworthy that osseointegration represents a protracted biological process, typically unfolding over extended durations ranging from weeks to months. The inherent inertness and modest bioactivity exhibited by PTa substrates may impede the expeditious advancement of this process. In response, researchers have undertaken endeavors to ameliorate these challenges by implementing diverse biomaterial coatings aimed at surface modification of PTa implants. Such interventions aim to bolster their bioactivity profile and foster enhanced integration with host bone tissue, thereby fostering their broader applicability and efficacy within clinical contexts.

Coating of biomaterials is a commonly used surface modification method that alters the surface properties of PTa surfaces by applying additional layers to them. These coatings include bioactive substances, cytokines, drugs, etc. to provide better cell attachment, enhance osteoinduction or inhibit bacterial infection. Common coating materials with bioactive substances include calcium phosphate (Cap), hydroxyapatite (HA), poly-lactic acid (PLA), and others. These coatings can improve the biocompatibility, bioactivity, and osteointegration of PTa, thereby facilitating bone defect repair.

### Bioactive substance-coated materials

**Fig. 3:**
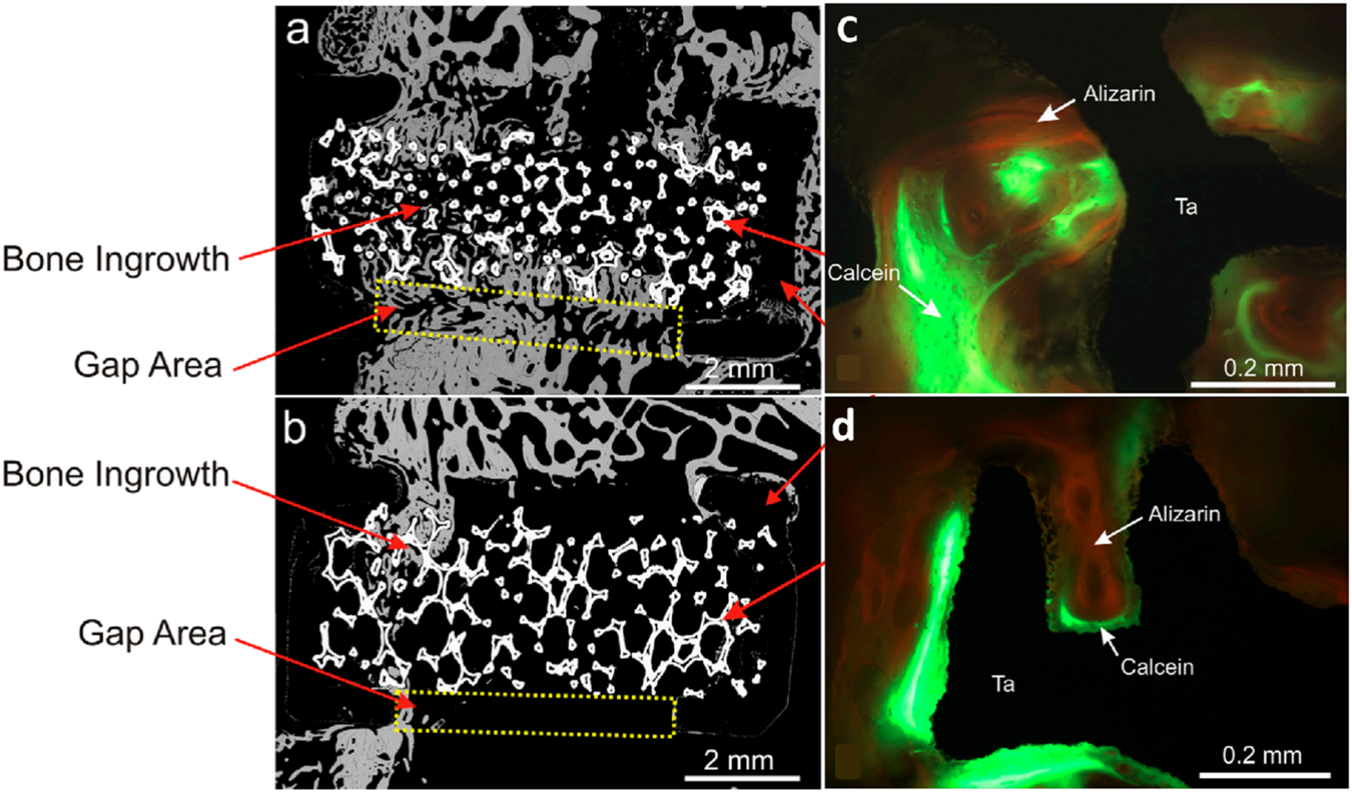
Backscattered electron micrographs showing gap filling and osseous ingrowth in relation to a PTa implant, both coated with (**a**) calcium phosphate and alendronate and (**b**) an uncoated counterpart. Representative patterns of new bone formation (**c**) On the uncoated porous tantalum (Ta) implant and (**d**) On the porous tantalum (Ta) implant coated with calcium phosphate and alendronate [55]. Reproduced under the terms of the Creative Commons Attribution 4.0 International (CC BY 4.0) license. Copyright © 2008 by the The Journal of Bone and Joint Surgery, Incorporated

Calcium phosphate (CaP) exhibits physicochemical characteristics akin to native bone tissue in vivo, demonstrating favorable osteoconductive properties conducive to intramatrix bone ingrowth [49]. Various techniques, including plasma spraying [50], magnetron sputtering [51], electrochemical deposition [52], ion-immersion injection [53], and sol-gel methodologies [54], have been employed to deposit CaP coatings onto substrates. These approaches aim to enhance the biomimetic attributes of matrices, thereby fostering augmented osseointegration and functional compatibility within biomedical applications. CaP has been leveraged for surface modification and as a medium for drug delivery onto PTa substrates. Garbuz et al. implemented alendronate-CaP coatings to address the interfacial discrepancy between PTa implants and bone tissue, effectively bridging the simulated bone defect. The augmentation of PTa implant surface bioactivity ensued upon the application of alendronate coating. This efficacious remedial process was attributed to the controlled release kinetics of alendronate, which locally impeded osteoclastic activity while potentiating osteoblastic function. Consequently, this orchestrated modulation facilitated enhanced osseous deposition onto the scaffold surface [55] (Fig. 3). Zhou et al. used the prepared Cap-polypropylene-acrylate (Cap-PLA) composites for coating Ta plates and porous scaffolds, and the experimental results showed that it was favorable for the adhesion and spreading of human osteoblast-like MG63 cells [56]. Xu et al. employed CaP and magnesium-doped CaP (Mg-CaP) coatings atop PTa scaffolds produced via selective laser melting (SLM) technology, utilizing a hydrothermal application method. Experimental outcomes evinced notable advancements in osteogenic efficacy with both coating variants, thereby markedly amplifying the osseointegrative capacity of PTa constructs [57]. García-Gareta et al. employed a concentrated simulated body fluid (SBF) solution to facilitate the deposition of amorphous CaP apatite-like particles onto Ta surfaces via a bionic rapid two-step immersion technique. This method was observed to foster the initial adhesion, proliferation, and osteogenic differentiation of bone marrow-derived mesenchymal stem cells (MSCs) [58]. Barrère et al. conducted an implantation study wherein CaP-coated PTa cylinders were surgically placed within the femoral diaphysis of 14 adult female goats. The investigation revealed a discernible augmentation in osseointegration, accompanied by notable observations of robust osteogenic activity [59].

**Fig. 4:**
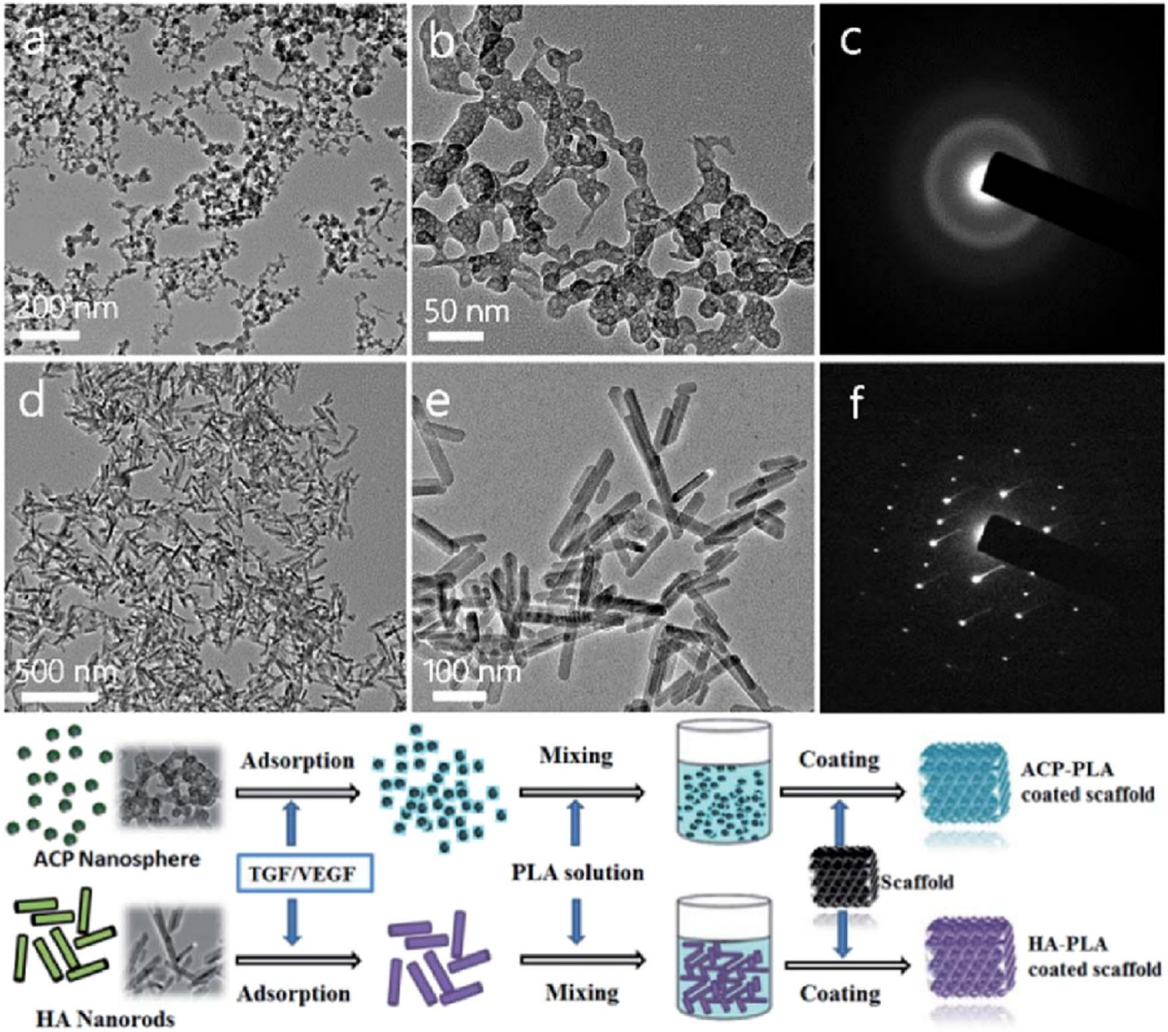
TEM micrographs and SAED patterns and Illustration of the strategy for the preparation (**a**, **b**) TEM micrographs of ACP nanospheres; (**c**) SAED pattern of ACP nanospheres; (**d**, **e**) TEM micrographs of HA nanorods; **f** SAED pattern of HA nanorods [61]. Reproduced under the terms of the Creative Commons Attribution 4.0 International (CC BY 4.0) license. Copyright © 2020 The Royal Society of Chemistry

The application of hydroxyapatite (HA) for surface refinement of PTa constructs has been documented. Within a canine experimental model, the utilization of zoledronic acid-hydroxyapatite (ZA-HA) coated PTa rods resulted in a notable augmentation of bone formation within the peri-implant milieu and internal interstices [60]. Zhou et al. prepared HA-PLA polymer-coated PTa scaffolds, which significantly improved the hydrophilicity of the structure as compared to bare and PLA-coated PTa scaffolds, and at the same time, osteoclast-like cells (MG63) on HA-PLA-coated PTa scaffolds exhibited good adhesion and spreading properties. In vivo, it repaired subchondral bone defects and formed a large amount of new bone, indicating that good results were obtained in bone defect repair [61] (Fig. 4). Antonio et al. elucidated that HA-modified Ta surfaces exhibit heightened bioactivity, consequently augmenting alkaline phosphatase activity. This enhancement was attributed to the intricate interplay between the surface morphology and chemical composition of HA coatings deposited onto Ta via plasma electrolytic oxidation (PEO) methodologies [62]. The HA coating doped with Ta not only promoted initial cell adhesion and accelerated proliferation, but also promoted osteogenic differentiation of BMSCs [63]. Wang et al. prepared bilayer-coated PTa scaffolds using micro-arc oxidation and hydrothermal treatment (HT), with HA nanorods/fibers as the outer layer and CaTa_2_O_6_ as the matrix as the inner layer The results demonstrated that osteoblastic cell survival and proliferation were significantly enhanced [64].

**Fig. 5:**
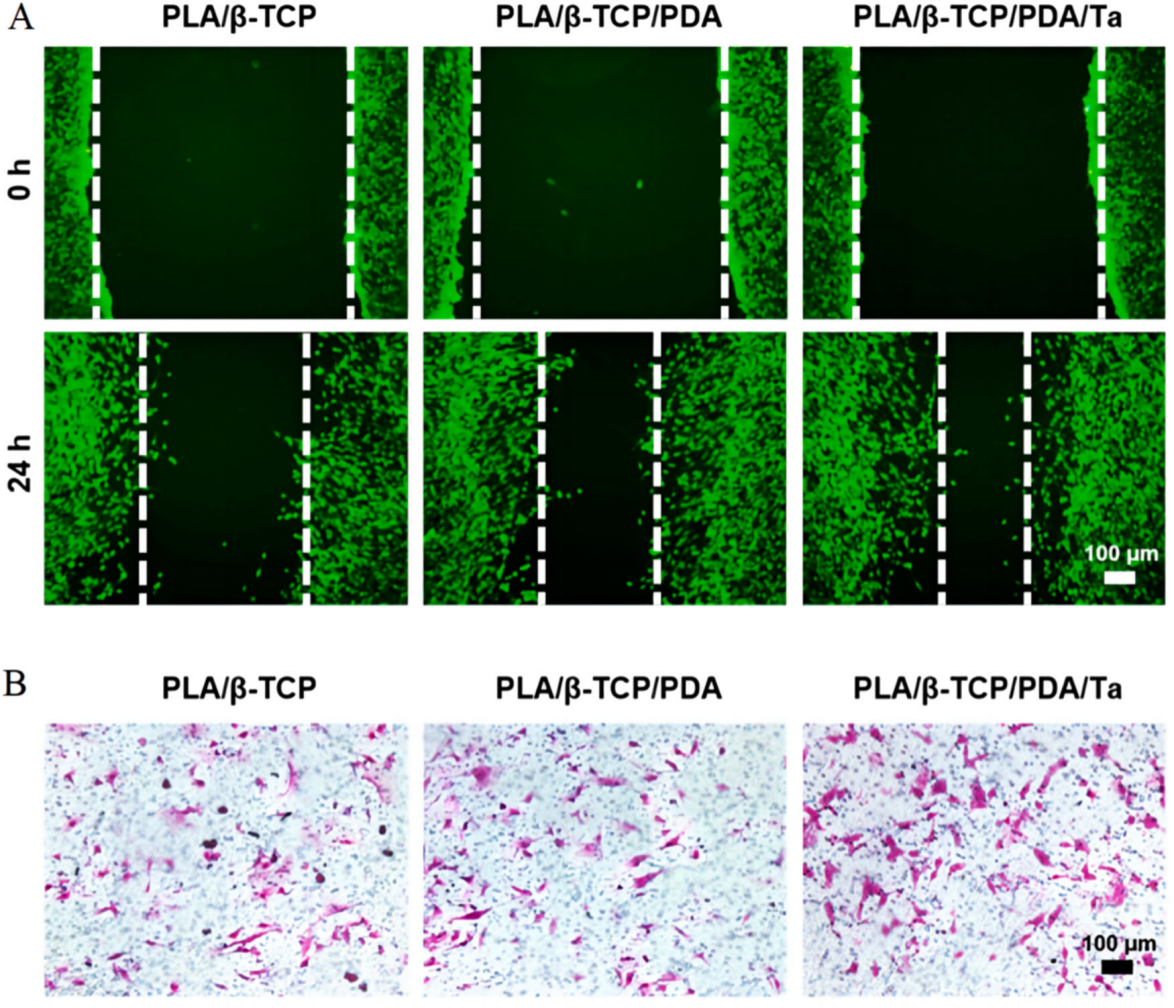
In vitro cell migration. **A** In vitro scratch assay of MC3T3-E1 cells. **B** Transwell assay of MC3T3-E1 cells [68]. Reproduced under the terms of the Creative Commons Attribution 4.0 International (CC BY 4.0) license. Copyright © 2022 Elsevier B.V

Poly-lactic acid (PLA), a prominent synthetic polymer, enjoys widespread application owing to its biodegradability, rendering it capable of substituting regenerated bone tissue over time. Its versatility extends to serving as a vehicle for drug delivery, a scaffold material in tissue engineering, and a constituent in bone repair formulations, among other applications [65, 66]. PLA demonstrates the capacity to enhance bone conductivity and foster bone formation, owing to its inherent biocompatibility and ability to degrade into non-toxic byproducts within the physiological milieu. Moreover, PLA exhibits a controlled degradation profile following its introduction into the human body, further enhancing its suitability for biomedical applications [67]. Liu et al. engineered porous scaffolds composed of PLA, beta-tricalcium phosphate (β-TCP), polydopamine (PDA), and Ta, characterized by favorable physical attributes. In vitro assessments underscored the scaffolds’ capacity to markedly stimulate cellular proliferation and migration while concurrently exhibiting osteogenic properties, thereby addressing the foundational requisites for bone regeneration and tissue mending [68] (Fig. 5). Zhou et al. devised a homogeneous suspension through the amalgamation of CaP nanospheres with PLA polymer, subsequently utilized for surface modification of PTa scaffolds. The incorporation of vascular endothelial growth factor (VEGF) and transforming growth factor (TGF) onto the scaffold was achieved. Their findings indicate that this composite scaffold offers a multifaceted approach, providing growth factor delivery, mechanical support, architectural guidance, and fostering subchondral bone regeneration [56]. The synergistic application of ion implantation alongside conventional magnetron sputtering represents a methodological advancement aimed at ameliorating the inherent hydrophobicity and biologically inert chemical composition of PLA. This dual treatment strategy engenders a transition towards a moderately hydrophilic surface characteristic, thereby fostering heightened interactions between cells and the material substrate. Moreover, in vivo assessment utilizing a rabbit distal femur implant model revealed pronounced enhancements in osseointegration and osteogenic responses, indicative of the therapeutic efficacy of the modified PLA construct [69]. Almansoori et al. fabricated a neuroconduit comprised of Ta and PLA, employing PLA as the outer layer and Ta as the inner layer. This construct demonstrated a notable capacity to promote peripheral nerve regeneration in vivo, while concurrently mitigating the formation of scar tissue within the regenerative milieu [70]. Hwang et al. conducted experiments to elucidate the effects of a Ta-PLA membrane on osteoblastic behavior, including attachment, proliferation, and differentiation. Their findings underscored a substantial enhancement in these cellular processes upon interaction with the Ta-PLA membrane. Furthermore, the demonstrated superior osteoconductive properties of Ta-PLA advocate for its potential utility as a membrane in guided bone regeneration protocols [71].

The Arg-Gly-Asp polypeptide (RGD) motif is prominently featured within extracellular matrix proteins, including fibronectin, osteoblastin, and salivary proteins, functioning as a key recognition site facilitating the binding of integrin receptors to extracellular ligands situated on cellular membranes. Gan et al. conducted an investigation wherein varying concentrations of RGD peptide (1 g/L, 5 g/L, and 10 g/L) were immobilized onto PTa sheets, each possessing a diameter of 10 mm and a thickness of 3 mm, via physical adsorption techniques. Their findings revealed an upregulation in the expression levels of osteoblast-specific protein Osteocalcin (OCN) and cytoskeletal protein F-actin relative to the unmodified control group. This augmentation underscores the advantageous role of RGD in bolstering osteoblast adhesion, facilitating cytoskeletal diffusion and reorganization upon the surface of PTa, thereby enhancing interfacial morphology and fostering osseointegration at the Ta-osteoblast nexus [72]. Wang et al. endeavored to enhance the surface properties of PTa by incorporating RGD motifs, subsequently applying these modified scaffolds in the remediation of segmental bone defects within rabbit radial models. Relative to unmodified counterparts, the RGD-functionalized PTa scaffolds elicited heightened bone formation both at the scaffold interface and within its internal porosity. Furthermore, biomechanical assessments revealed superior mechanical properties in the RGD-modified PTa cohort compared to the unmodified scaffold group [73]. McNichols et al. have demonstrated the influence of surface topography and functionalization strategies on Ta substrates, particularly through the incorporation of cyclic-(arginine-glycine-aspartic acid-d-phenylalanine-lysine) (cRGDfK). Their findings delineate the profound impact of these modifications on endothelial cell behaviors, including attachment, diffusion, and proliferation dynamics. Notably, augmented surface properties engendered by cRGDfK functionalization correlate with elevated cell density, enhanced diffusion kinetics, and increased intercellular interactions, underscoring the pivotal role of surface characteristics in modulating endothelial cell responses [74]. Mas-Moruno et al. elucidated the effects of surface functionalization on Ta substrates through the incorporation of RGD motifs. Their investigation revealed that RGD immobilization via physical adsorption exerted a pronounced influence on the attachment and spreading behaviors of osteoblast-like cells on Ta surfaces. This augmentation underscores the role of surface chemistry in modulating cellular responses, particularly in facilitating initial cell adhesion and spreading, crucial processes in tissue integration and regeneration [75].

### Cytokine

Bone morphogenetic protein 7 (BMP-7), belonging to the bone morphogenetic protein family, exhibits potent osteoinductive properties and serves as a pivotal regulator in bone development, defect remediation, and cartilage differentiation processes. Since its initial application in 2001, BMP-7 has been instrumental in promoting osteogenic and chondrogenic differentiation pathways, notably by inducing mesenchymal stem cell commitment towards the osteoblastic and chondrocytic lineages. This multifaceted functionality underscores its significance in regenerative medicine and underscores its therapeutic potential in addressing musculoskeletal pathologies [76, 77]. Wang et al. conducted experimental investigations wherein BMP-7-coated PTa scaffolds were compared against their uncoated counterparts in promoting tissue regeneration post-surgery. Their findings revealed marked enhancements in cartilage and bone formation within the BMP-7-coated group, characterized by increased volume fraction of newly formed bone, augmented quantity and quality of bone trabeculae, and elevated maximum bone release force relative to the uncoated scaffold. Micro-computed tomography (micro-CT) examinations and histological assessments further corroborated these observations, illustrating heightened bone tissue deposition within the scaffold interstices in the BMP-7-coated cohort. Moreover, biomechanical analyses demonstrated a significantly enhanced maximum pulling force in the coated group, underscoring the biomechanical superiority conferred by BMP-7 functionalization in Ta scaffolds [78]. Zhang et al. conducted a study involving the inoculation of second-generation chondrocytes isolated from 3-week-old New Zealand rabbits onto PTa scaffolds, followed by the addition of BMP-7 to create a BMP-7/Ta/chondrocyte composite. Their investigation revealed a substantial augmentation in chondrocyte proliferation and extracellular matrix production in vitro within this composite system. Furthermore, they observed a notable upregulation in the expression of key chondrogenic markers including Col-II, AGG and Sox9 levels, indicative of enhanced chondrogenic differentiation potential elicited by the BMP-7-treated Ta-chondrocyte construct [79]. Bone morphogenetic protein 2 (BMP-2), sanctioned by the Food and Drug Administration (FDA) for its efficacy in bolstering bone regeneration, stands as a paramount growth factor renowned for its pivotal role in fostering osteoblast differentiation and precipitating osteogenesis. Renowned for its regulatory prowess in orchestrating bone formation processes, BMP-2 occupies a central position in the realm of osteogenic induction, serving as a cornerstone in therapeutic interventions aimed at augmenting skeletal tissue repair and regeneration [80–82]. Yu et al. found that BMP-2-functionalized PTa enhanced osteogenic differentiation of MC3T3-E1 cells [83].

### Drugs

**Fig. 6:**
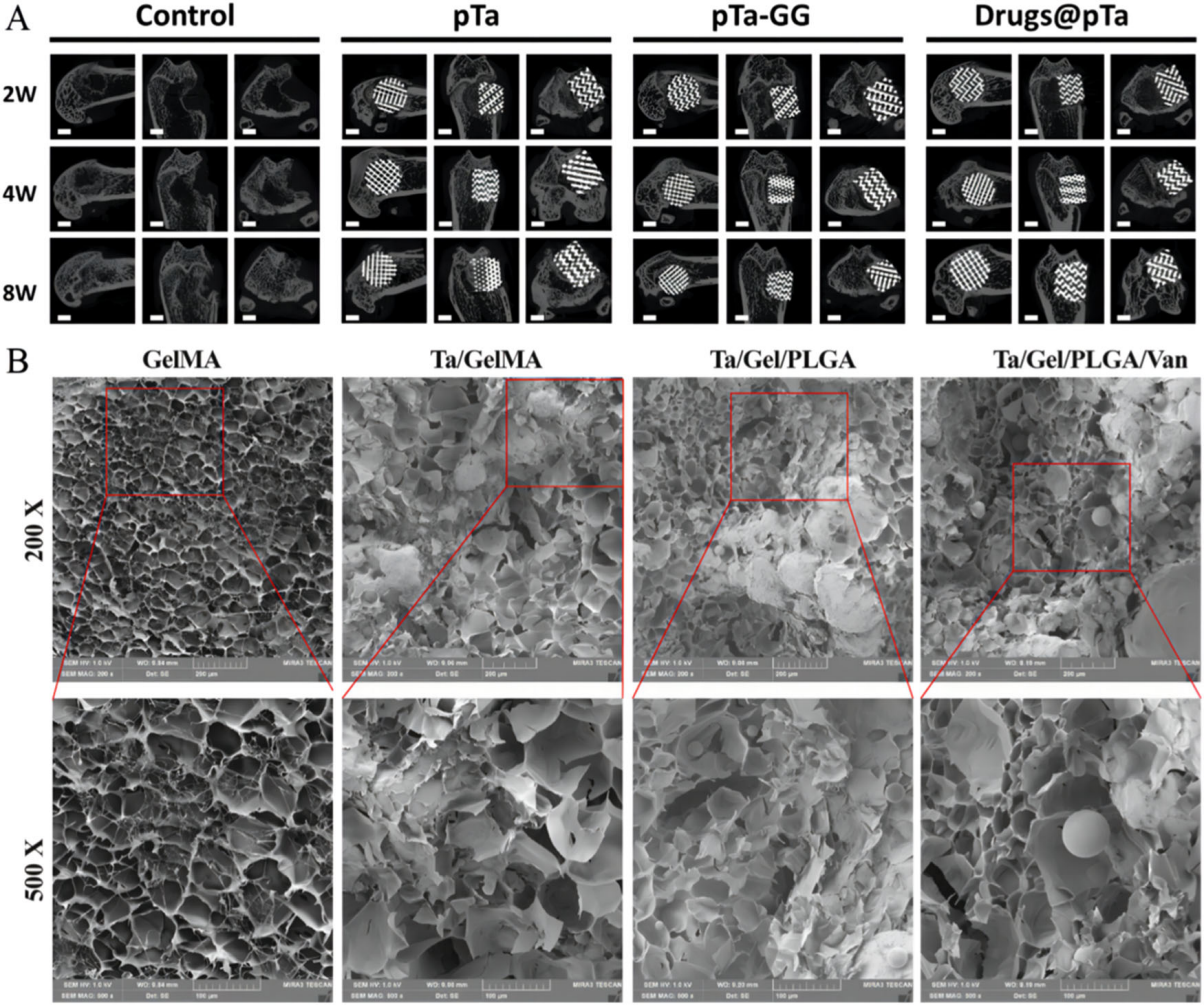
**A** In vivo osteogenesis of Ta-loaded anti-tuberculosis drugs, CT results of rats in control group, PTa group, PTa-GG group and Drugs@PTa group (2w, 4w and 8w) [84] Reproduced under the terms of the Creative Commons Attribution 4.0 International (CC BY 4.0) license.Copyright © 2021 Wiley-VCH GmbH. **B** Representative SEM image of the Ta/Gel/PLGA/Van composite scaffolds [86]. Reproduced content is open access. Copyright© 2023 The Authors. Published by KeAi Communications Co., Ltd

PTa loaded with drugs can fulfill the requirements of good bone biomechanics, cytocompatibility and antimicrobial function. Hua et al. devised a sophisticated strategy by amalgamating PTa with the anti-tuberculosis agents isoniazid and rifampicin, supplemented by surface modification with polydopamine to enhance adhesion properties. This meticulous approach facilitated sustained drug release kinetics both in vitro and in vivo, thereby prolonging the antimicrobial efficacy of the composite bioscaffold. Remarkably, the engineered bioscaffold exhibited potent inhibition against Staphylococcus aureus proliferation, while maintaining favorable biocompatibility profiles and fostering unimpeded osteogenic differentiation of induced rat bone marrow mesenchymal stem cells. The concurrent achievement of protracted local drug release kinetics alongside facilitated bone regeneration underscores the potential of this composite system as a versatile platform for targeted and sustained therapeutic interventions in complex osseous infections [84] (Fig. 6A).

Rodríguez-Contreras et al. developed antimicrobial coatings for Ta implants using polyhydroxystreptanate (PHA) as a matrix carrying the active ingredient, and gentamicin coated PTa structures, especially the inner surfaces, by dip-coating technique. A homogeneous biopolymer coating that was non-toxic and well biointegrated was obtained and showed good antimicrobial properties [85].

Qian et al. studied the production of Ta/GelMA/PLGA/Van composite scaffolds for the repair of infected bone defects by encapsulating vancomycin (Van) into poly(lactic acid-co-glycolic acid) (PLGA) microspheres using a liquid method, and then loading them into additively fabricated PTa (AM-Ta) by gelatinized methacryloylamide (GelMA) hydrogel, which was demonstrated to have good biocompatibility, as well as bacteriostatic and osseointegrative properties [86] (Fig. 6B). Sautet et al. demonstrated in vitro that PTa containing Van had higher Van concentrations than the lowest inhibitory concentration of S. aureus in all groups at day 3 [87]. If present in vivo, to a similar extent, this intrinsic property may be useful in preventing and/or treating periprosthetic joint infections. Liu et al. prepared PTa scaffolds loaded with Van by a combination of chemical grafting and electrostatic assembly techniques. In vitro experiments showed that the scaffolds could rapidly kill initially adhering bacteria and effectively prevent biofilm formation. In vivo experiments demonstrated that the scaffolds showed effective bacterial clearance and inflammation control in soft tissues and created an immune microenvironment suitable for early tissue repair [88].

Guo et al. employed a sophisticated approach to engineer PTa implants, leveraging electrostatic self-assembly techniques to immobilize a composite coating comprising hyaluronic acid, methylated collagen, and a ternary copolymer with polyelectrolytes onto the implant surface. This surface modification facilitated the loading of the anticancer agent adriamycin onto the PTa matrix, thereby endowing the implant with sustained drug delivery capabilities. The study revealed a prolonged release profile, with drug elution persisting for up to one month. Notably, a zero-order release kinetics was achieved by the second day, reaching peak concentrations (5.64 g/mL) after 6 h, and maintaining therapeutic levels (>0.25 g/mL) for an extended duration thereafter. Importantly, all released drug formulations exhibited significant inhibitory effects on the proliferation of the chondrosarcoma cell line SW1353, underscoring the potential of this engineered system for targeted and sustained chemotherapy in osseous malignancies [89].

Drug-laden biomaterials serve dual purposes, serving not only as adjunctive fillers in bone defect reconstruction procedures but also as vehicles for localized and protracted drug administration in the management of osseous neoplasms. Nonetheless, the efficacy of Ta-based drug-eluting coatings remains encumbered by challenges pertaining to drug release kinetics, dosage control modulation, and the sustainability of antimicrobial actions over extended durations. Additionally, a paucity of investigations exists regarding the potential emergence of drug resistance following the deployment of Ta-coated antibiotic implants.

## Conclusion and future prospect

PTa has emerged as a cornerstone in bone tissue engineering, owing to its notable attributes including biocompatibility, corrosion resistance, and biomechanical properties mirroring those of native bone tissue. Its inherent osteogenic potential facilitates efficacious bone regeneration within defect sites, yielding clinically favorable outcomes. Nevertheless, there persists a demand for refining the characteristics of PTa implants. The advent of 3D printing technology has ushered in a new era, offering unparalleled avenues for the personalized design and fabrication of PTa-based implants. Through meticulous adjustments to macrostructural parameters, pore morphology, and porosity, 3D printed PTa implants hold promise in addressing the diverse needs of patients, particularly those confronted with intricate load-bearing bone defects.

Moreover, to augment the biological potency of PTa, diverse methodologies have been employed to modify and enhance its functional attributes. Nonetheless, extant investigations have predominantly unfolded within controlled laboratory settings. The array of surface modification techniques applied to Ta presents multifaceted avenues for interrogating Ta interfaces. Presently, the clinical integration of modified PTa remains suboptimal, marked by a paucity of extensive, multicenter, long-term clinical follow-up data, thereby impeding a comprehensive assessment of the safety and efficacy of PTa bone implants. At this juncture, a majority of studies pertaining to PTa surface modification are confined to in vitro investigations or short-term in vivo animal models. However, as advancements in technology progress, the potential emerges for concurrent loading of various pharmacological agents, cytokines, or cells onto PTa scaffolds, thereby accommodating a spectrum of functional exigencies. Such versatility positions PTa as a potential therapeutic candidate across a gamut of pathological conditions, including osteoporosis, infections, diabetes, and even neoplasms. Continued inquiry is warranted to fully elucidate the expansive therapeutic utility of PTa, with the anticipation that its clinical repertoire will continue to burgeon.
